# A Transcription Factor *SlNAC10* Gene of *Suaeda liaotungensis* Regulates Proline Synthesis and Enhances Salt and Drought Tolerance

**DOI:** 10.3390/ijms23179625

**Published:** 2022-08-25

**Authors:** Xinran Du, Mingxing Su, Yang Jiao, Suxiang Xu, Jieqiong Song, Hongfei Wang, Qiuli Li

**Affiliations:** Key Laboratory of Plant Biotechnology of Liaoning Province, School of Life Sciences, Liaoning Normal University, Dalian 116081, China

**Keywords:** *Suaeda liaotungensis*, NAC transcription factor, abiotic stress, proline, transgenic *Arabidopsis*

## Abstract

The NAC (NAM, ATAF1/2, and CUC2) transcription factors are one of the largest families of transcription factors in plants and play an important role in plant development and the response to adversity. In this study, we cloned a new NAC gene, *SlNAC10*, from the halophyte *Suaeda liaotungensis* K. The gene has a total length of 1584 bp including a complete ORF of 1107 bp that encodes 369 amino acids. The *Sl*NAC10-GFP fusion protein is located in the nucleus and *Sl*NAC10 has a transcription activation structural domain at the C-terminus. We studied the expression characteristics of *SlNAC10* and found that it was highest in the leaves of *S. liaotungensis* and induced by drought, salt, cold, and abscisic acid (ABA). To analyze the function of *SlNAC10* in plants, we obtained *SlNAC10* transgenic *Arabidopsis*. The growth characteristics and physiological indicators of transgenic *Arabidopsis* were measured under salt and drought stress. The transgenic *Arabidopsis* showed obvious advantages in the root length and survival rate; chlorophyll fluorescence levels; and the antioxidant enzyme superoxide dismutase (SOD), peroxidase (POD), and catalase (CAT) activities, and the proline content was higher than that of the wild-type (WT) *Arabidopsis*, whereas the relative electrolyte leakage and malondialdehyde (MDA) content were lower than those of the wild-type *Arabidopsis*. We explored the regulatory role of *Sl*NAC10 on proline synthesis-related enzyme genes and found that *Sl*NAC10 binds to the AtP5CS1, AtP5CS2, and AtP5CR promoters and regulates their downstream gene transcription. To sum up, *Sl*NAC10 as a transcription factor improves salt and drought tolerance in plants possibly by regulating proline synthesis.

## 1. Introduction

In recent years, with increasingly serious environmental pollution, plants have been subjected to more and more abiotic stresses, which have seriously affected their growth and development. Upon sensing a stress signal, plants increase their resistance to the stressful environment. Transcription factors (TFs) play a key role in the conversion of stress signal perception into stress-responsive gene expression during signal transduction processes [1]. TFs make up approximately 7% of the coding part of the plant transcriptome. A typical TF is comprised of four parts: a conserved DNA-binding part, a variable transcriptional regulatory part, an oligomeric part, and a nuclear localization signal (NLS) for protein entry into the nucleus [2]. In plants, TFs usually belong to large gene families such as the NAC (no apical meristem (NAM), *Arabidopsis* transcriptional activator factor (ATAF), cup-shaped cotyledon (CUC)) superfamily [3]. Plant NAC proteins contain a highly conserved DNA-binding (NAC) domain at the N-terminal and a variable transcriptional regulatory domain at the C-terminal [2]. The N terminus consists of nearly 160 amino acid residues divided into five major conserved sub-regions named A to E [4].

So far, many studies have demonstrated the involvement of the NAC transcription factors in plant growth and development including in secondary wall formation [5,6], lateral root development [7,8], seed germination [9], organ formation [10,11], plant senescence [12,13], phytohormone signaling [14,15], and immune responses [16]. In addition, an increasing number of studies have explored the functions of the NAC family in response to various abiotic stresses. For example, *SlNAC1* and *SlNAM1* were induced by salt stress in the salt-tolerant tomato cultivar, Edkawi, whereas the expression pattern was different in a salt-sensitive cultivar, ZS-5. *SlNAC1* and *SlNAM1* may play an important role in the stress tolerance of tomatoes [17]. Overexpression of the *AtJUB1* gene of *Arabidopsis thaliana* in *Solanum lycopersicum cv.* MoneyMaker (tomato) was found to partially alleviate water deficit stress during the vegetative stage [18]. *BoNAC019* is a gene isolated from cabbage (*Brassica oleracea*) that is homologous to *AtNAC019*. *BoNAC019* may be involved in the regulation of drought resistance by inducing ABA catabolism genes and reducing ABA content [19]. In rice, ONAC066 is a positive regulator of drought and oxidative stress tolerance [20] and OsNAP acts as a transcriptional activator in response to abiotic stress [21]. *GmNAC2*-overexpressing tobacco lines were highly sensitive to drought, high-salt, and low-temperature stresses [22] and overexpression of *GmNAC11* and *GmNAC20* improved salt tolerance in transgenic plants, whereas overexpression of *GmNAC20* in plants increased tolerance to cold damage [23] and transgenic plants overexpressing *GmNAC085* exhibited slight growth retardation but improved drought tolerance [24]. The *VvNAC17* gene is expressed in different tissues of the grapevine after drought, high temperatures, freezing, and SA and ABA treatments, and *VvNAC17* overexpression in *Arabidopsis* improved tolerance to environmental stress [25]. Salt treatment significantly induced the expression of the apple *MdNAC047* gene, which improved plant tolerance to salt stress and promoted ethylene release [26]. NAC genes named *CaNAC064* and *CaNAC035* were isolated from the pepper. The MDA content, chilling injury index, and relative electrolyte leakage were lower in *CaNAC064*-overexpressing *Arabidopsis* than in wild plants under low-temperature stress and CaNAC064 positively regulated plants’ cold tolerance. *CaNAC035*overexpressing *Arabidopsis* plants showed higher germination rates and fresh weight after mannitol and NaCl treatment; *CaNAC035* is a positive regulator of abiotic stress tolerance in pepper and it acts through multiple signaling pathways [27,28]. Overexpression of the *CiNAC3* and *CiNAC4* genes from *Caragana Intermedia* reduced ABA sensitivity during seed germination and improved salt tolerance in transgenic *Arabidopsis* [29]. When the *SlNAC2* gene of *Solanum lycopersicum* L. was overexpressed in *Arabidopsis*, soil stress analysis showed that transgenic plants survived better under both salt and drought stress, and had a better antioxidant response; it also reduced the accumulation of reactive oxygen molecules and retained better water in tissues [30]. Other studies on maize have similarly shown that the expression of the NAC genes *ZmSNAC1* and *ZmNAC55* were induced by low temperatures, high salt, drought stress, and abscisic acid treatment. *ZmSNAC1* was a stress-response transcription factor in the positive regulation of abiotic stress tolerance. Overexpression of *ZmNAC55* in *Arabidopsis* led to high sensitivity to ABA during germination but enhanced drought tolerance compared to wild-type seedlings [31,32].

*Suaeda liaotungensis*, an annual herb under the genus *Suaeda* in Chenopodiaceae, is a saline plant living in a saline coastal environment. The NAC transcription factors of glycophytes have been extensively studied but the functions of the NAC transcription factors of halophytes are yet to be discovered. In our previous study, we found that *SlNAC1*, *SlNAC2*, *SlNAC7*, and *SlNAC8* from *S. liaotungensis* were involved in the plant response to abiotic stress and significantly enhanced the tolerance of transgenic plants to stressful environments [33,34,35,36]. In this study, we identified another *S. liaotungensis* NAC transcription factor named *SlNAC10* (*Suaeda liaotungensis*, NAM (No apical meristem), ATAF1/2 (*Arabidopsis* transcriptional activator factor), CUC (cup-shaped cotyledon), NO. 10 gene). Firstly, we cloned the *SlNAC10* gene and performed bioinformatics analysis. Subsequently, we found that the *Sl*NAC10 protein was localized in the nucleus and had transcriptional activation. The transformation of the *SlNAC10* gene into *Arabidopsis thaliana* revealed that the transgenic plants had a significant survival advantage under salt and drought stress compared to wild-type plants. In addition, proline content was increased in *SlNAC10* transgenic *Arabidopsis* under unfavorable conditions so we investigated the role of *SlNAC10* in the regulation of genes related to proline synthesis and found that *Sl*NAC10 could bind to the promoters of the *AtP5CS1*, *AtP5CS2,* and *AtP5CR* genes and regulate the transcription of their downstream genes. The above findings suggest that the SlNAC10 transcription factor enhanced plant stress tolerance by regulating proline synthesis, which helps us to further study the NAC transcription factor and its stress tolerance mechanism in salt-tolerant plants of *S. liaotungensis*.

## 2. Results

### 2.1. SlNAC10 Encodes a Protein Containing the NAC Domain

**Figure 1:**
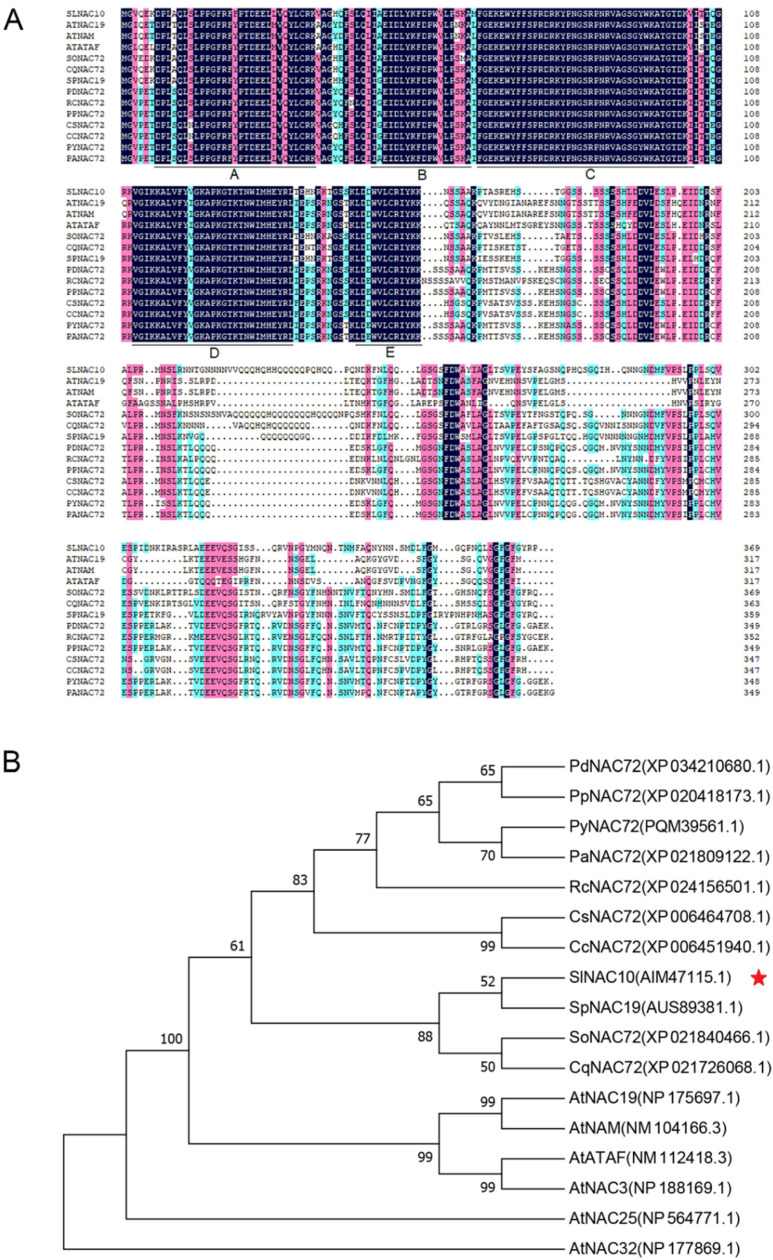
Multiple alignments and phylogenetic tree of *Sl*NAC10 with other NAC proteins. (**A**) Comparison of *Sl*NAC10 protein with NAC proteins from other plant species. Residues that are conserved in all proteins are highlighted. The five subdomains of the NAC domain are denoted by lines designated with the letters A–E. (**B**) Evolutionary tree analysis of *Sl*NAC10 (indicated by a red star) and other NAC proteins. The accession numbers are as follows: SlNAC10 (*Suaeda liaotungensis*, AIM47115.1), PdNAC72 (*Prunus dulcis*, XP_034210680.1), PpNAC72 (*Prunus persica*, XP_020418173.1), PyNAC72 (*Prunus yedoensis var. nudiflora*, PQM39561.1), PaNAC72 (*Prunus avium*, XP_021809122.1), RcNAC72 (*Rosa chinensis*, XP_024156501.1), CsNAC72 (*Citrus sinensis*, XP_006464708.1), CcNAC72 (*Citrus clementina*, XP_006451940.1), SpNAC19 (*Sesuvium portulacastrum*, AUS89381.1), SoNAC72 (*Spinacia oleracea*, XP_021840466.1), CqNAC72 (*Chenopodium quinoa*, XP_021726068.1), AtNAC19 (*Arabidopsis thaliana*, NP_175697.1), AtNAM (*Arabidopsis thaliana*, NM_104166.3), AtATAF (*Arabidopsis thaliana*, NM_112418.3), AtNAC3 (*Arabidopsis thaliana*, NP_188169.1), AtNAC25 (*Arabidopsis thaliana*, NP_564771.1), AtNAC32 (*Arabidopsis thaliana*, NP_177869.1).

Based on the EST sequence of *SlNAC10* in the transcriptome database, the cDNA sequence of *SlNAC10* (GenBank NO. KJ933532.1) was obtained by PCR amplification and 3′ and 5′ RACE. *SlNAC10* has a total length of 1584 bp, including a complete ORF of 1107 bp that encodes 369 amino acids, with ATG as the start codon and TAG as the stop codon. Conserved domains program analysis showed that *SlNAC10* had a conserved NAM domain containing the typical five subdomains of A, B, C, D, and E (Figure 1A). The *SlNAC10* sequence was analyzed using the Blastn program on the NCBI (http://www.ncbi.nlm.nih.gov, accessed on 21 May 2022) website, and a phylogenetic tree was constructed using MEGA 7.0. *Sl*NAC10 had a high similarity to *At*NAC19, *At*NAM, and *At*ATAF, which suggested that *Sl*NAC10 belonged to the NAM subfamily (Figure 1B).

### 2.2. SlNAC10 Is a Stress-Responsive Gene

**Figure 2:**
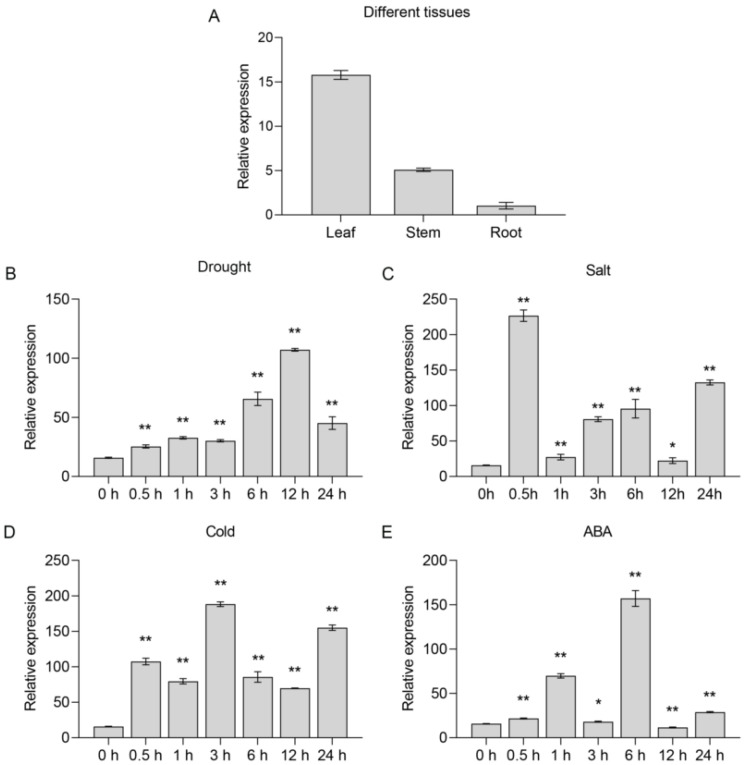
Expression patterns of *SlNAC10* in *S. liaotungensis* under normal conditions. (**A**) Expression patterns of *SlNAC10* in roots, leaves, and stems under normal conditions. Expression patterns of *SlNAC10* in seedling leaves in response to (**B**) salt treatment; (**C**) drought treatment; (**D**) cold treatment; (**E**) ABA treatment. The 2^−ΔΔCT^ method was used to measure relative expression levels of the target gene. Each bar represents the mean ± SD of triplicate experiments (* *p* < 0.05; ** *p* < 0.01).

The expression levels of *SlNAC10* in several organs of *S. liaotungensis*, as well as the response to various abiotic stressors and ABA stimulation, were determined by qRT-PCR. We found that the *SlNAC10* gene was expressed in different organs, with the highest expression in leaves (Figure 2A). Under drought stress, *SlNAC10* gene expression was highest at 12 h (Figure 2B); under salt stress, expression was significantly higher at 0.5 h and 24 h (Figure 2C); under low-temperature stress at 4 °C, expression was highest at 3 h and higher at 24 h (Figure 2D); and under ABA treatment, expression was significantly higher at 6 h than other periods (Figure 2E). In response to drought, high salt, low-temperature stress, and ABA treatment, *SlNAC10* was recognized as a stress-responsive gene.

### 2.3. SlNAC10 Is Located in the Nucleus and Has Transcriptional Activation Activity

**Figure 3:**
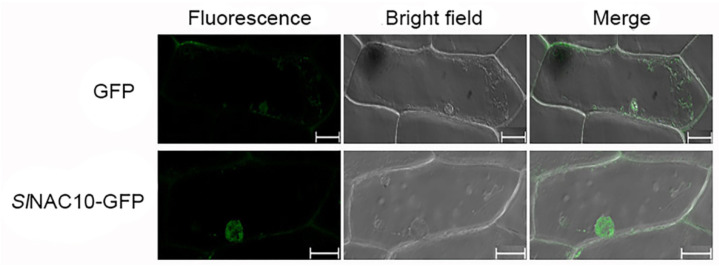
Subcellular localization of *Sl*NAC10 fused with GFP. GFP and *Sl*NAC10-GFP were expressed transiently in onion epidermal cells and observed using confocal microscopy. Fluorescence, green fluorescence image; Merge, merged images of bright field and fluorescence. The scale bar corresponds to 50 μm.

The pEGAD-*Sl*NAC10-GFP and pEGAD-GFP plasmids were injected into onion epidermal cells for transient expression. The *Sl*NAC10-GFP protein was found only in the nucleus under laser confocal microscopy with 488 nm excitation light, whereas the GFP protein produced by the control pEGAD vector was found in the nucleus and cytoplasm of the cells (Figure 3). The results indicated that the *Sl*NAC10 protein was localized in the nucleus.

**Figure 4:**
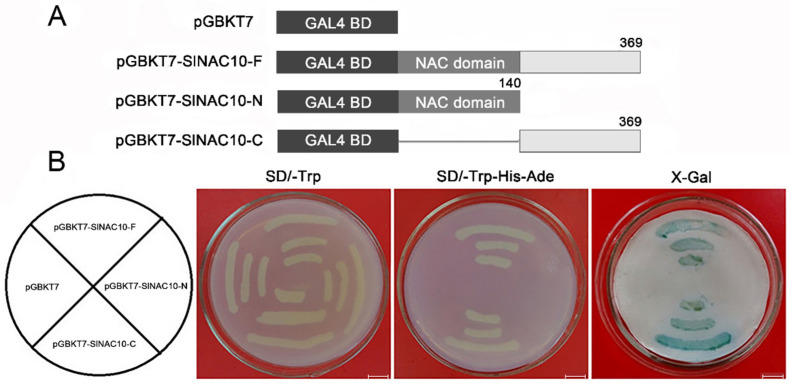
Trans-activational analysis of *Sl*NAC10 in yeast. (**A**) Schematic diagrams of the pGBKT7 vector, full-length (pGBKT7-*SlNAC10*-F), N-terminal region (pGBKT7-*SlNAC10*-N), and C-terminal region (pGBKT7-*SlNAC10*-C) of the *Sl*NAC10 protein, which were fused to the GAL4 DNA-binding domain. The numbers indicate the positions of the amino acids. (**B**) Growth on the selective medium of yeast cells transformed with different constructs using pGBKT7 as a control. The *β*-galactosidase activity was determined using X-Gal as a substrate. The scale bar corresponds to 1 cm.

The full-length coding sequence, N-terminal domain, and C-terminal domain of *SlNAC10* were inserted into the pGBKT7 vector (Figure 4A) and then the pGBKT7-*SlNAC10*-F (full length), pGBKT7-*SlNAC10*-N (N-terminal), pGBKT7-*SlNAC10*-C (C-terminal), and pGBKT7 empty vectors were transformed into yeast cells AH109. Each yeast cell grew normally on an SD/-Trp medium. Only pGBKT7-*SlNAC10*-F and pGBKT7-*SlNAC10*-C were able to grow normally on the SD/-Trp-His-Ade medium. *β*-galactosidase activity displayed a blue color for pGBKT7-*SlNAC10*-F and pGBKT7-*SlNAC10*-C (Figure 4B). The above results indicate that *Sl*NAC10 has a transcriptional activation role and the transcriptional activation domain is at the C-terminus.

### 2.4. Overexpression of SlNAC10 in Transgenic Arabidopsis Thaliana Improves Salt and Drought Tolerance

After kanamycin screening, PCR, and RT-PCR identification (Figure S1), T3 homozygous transgenic *Arabidopsis* lines were obtained. Therefore, *SlNAC10* high expression lines (L1, L2), wild-type (WT), and pBI121 control vector lines (VT1, VT2) were selected for morphological and physiological characterization detection.

**Figure 5:**
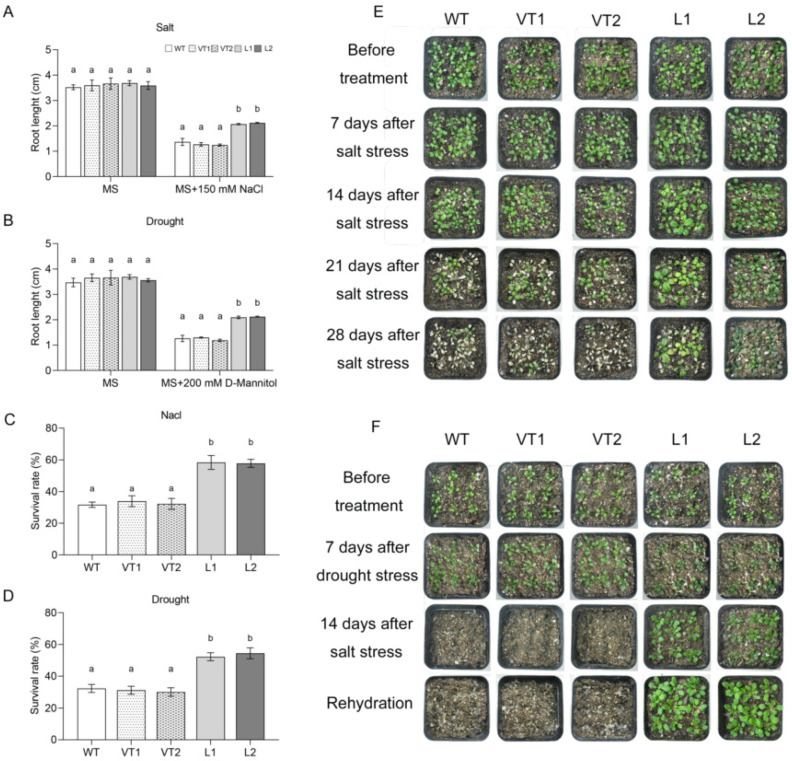
The root lengths and survival rates of *Arabidopsis* under drought and salt stress. The primary root length of wild and transgenic *Arabidopsis* cultured in (**A**) MS and MS + 150 mM NaCl; (**B**) MS and MS + 200 mM D-mannitol. The survival rates of wild and transgenic *Arabidopsis* under (**C**) MS + 150 mM NaCl; (**D**) MS+200 mM D-mannitol. Phenotypes of each line of *Arabidopsis* at different times of (**E**) salt treatment; (**F**) drought treatment. The data were means ± SD from three independent replications. Different lowercase letters indicate significant differences between different columns under the same treatment conditions (*p* < 0.05).

Root length and survival rates are often used as indicators of plant response to abiotic stresses. The determination of the root length of *Arabidopsis* seedlings grown on different MS media showed that the primary root lengths of all *Arabidopsis* seedlings were essentially the same when cultured in normal MS; in MS + 150 mM NaCl and MS + 200 mM D-mannitol, the primary root lengths of both the L1 and L2 lines were longer than those of the VT1, VT2, and WT lines (Figure 5A,B). The survival rate of *Arabidopsis thaliana* under different treatments showed that the L1 and L2 lines survived significantly better than the VT1, VT2, and WT lines under salt stress treatment. The leaves of the VT1, VT2, and WT lines showed a loss of green color at 21 d of treatment and the VT1, VT2, and WT lines showed extensive mortality at 28 d of treatment with a survival rate of only about 30% (Figure 5C,E). Under drought treatment, the leaves of *Arabidopsis* thinned and slowly deepened in color as the stress time increased and then began to shrivel and even die. The results of the survival rate of the lines after 3 d of rehydration showed that the L1 and L2 lines had a survival rate of over 50%, whereas only about 30% of the seedlings of the VT1, VT2, and WT lines continued to grow (Figure 5D,F).

**Figure 6:**
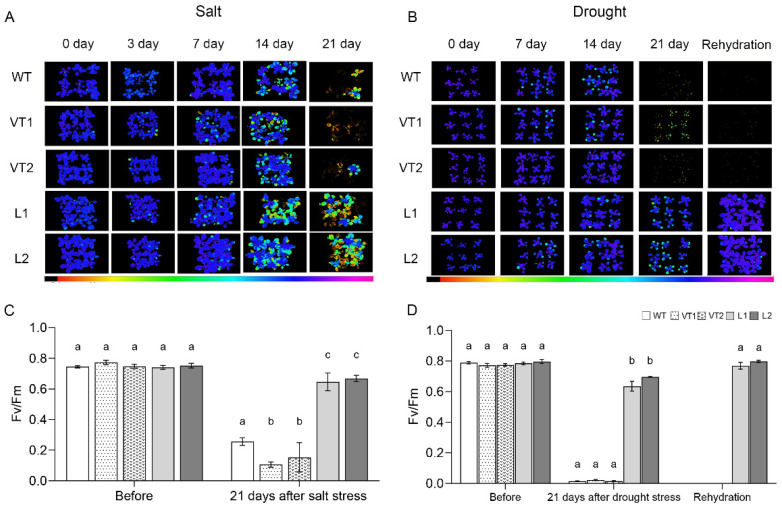
Chlorophyll fluorescence analysis of wild and transgenic *Arabidopsis* under stress. Chlorophyll fluorescence imaging of wild and transgenic *Arabidopsis* under (**A**) salt treatment; (**B**) drought treatment. Blue light means that the photosynthesis of the plant is proceeding normally, yellow light means that the photosynthesis of the plant is affected. IMAGING-PAM measures fluorescence imaging on an area of 10 × 13 cm. Column statistics of Fv/Fm values for wild and transgenic *Arabidopsis* under (**C**) salt treatment; (**D**) drought treatment. The data were means ± SD from three independent replications. Different lowercase letters indicate significant differences between different columns under the same treatment conditions (*p* < 0.05).

Chlorophyll fluorescence analysis is an effective, non-destructive technique used to measure the photochemical efficiency of PSII; Fv/Fm (chlorophyll fluorescence emission) estimates the maximum potential light energy utilization of PSII and fluorescence imaging of Fv/Fm gives a clear indication of the extent of the damage to the plant. Before the stress treatment, all *Arabidopsis* lines showed blue chlorophyll fluorescence imaging and Fv/Fm values between 0.7 and 0.8, indicating that the *Arabidopsis* plants were not damaged. After 7–14 d of salt treatment, the chlorophyll fluorescence of *Arabidopsis* leaves changed from blue to yellow and the Fv/Fm values decreased to varying degrees in all lines. After 21 d of salt treatment, chlorophyll fluorescence imaging of the L1 and L2 lines showed some plant damage but the Fv/Fm values were as high as 0.6–0.7. However, most plants of the WT, VT1, and VT2 lines lost chlorophyll fluorescence and the Fv/Fm values were only between 0.1 and 0.2 and the plants were largely dead (Figure 6A,C). The chlorophyll fluorescence of the WT, VT1, and VT2 lines gradually disappeared with the extension of the drought treatment time and the Fv/Fm values decreased significantly. After rehydration, the L1 and L2 lines still showed blue chlorophyll fluorescence imaging and a slight increase in the Fv/Fm values, whereas the WT, VT1, and VT2 lines did not recover chlorophyll fluorescence and the Fv/Fm values were almost all zero, indicating that *Arabidopsis* had died (Figure 6B,D).

**Figure 7:**
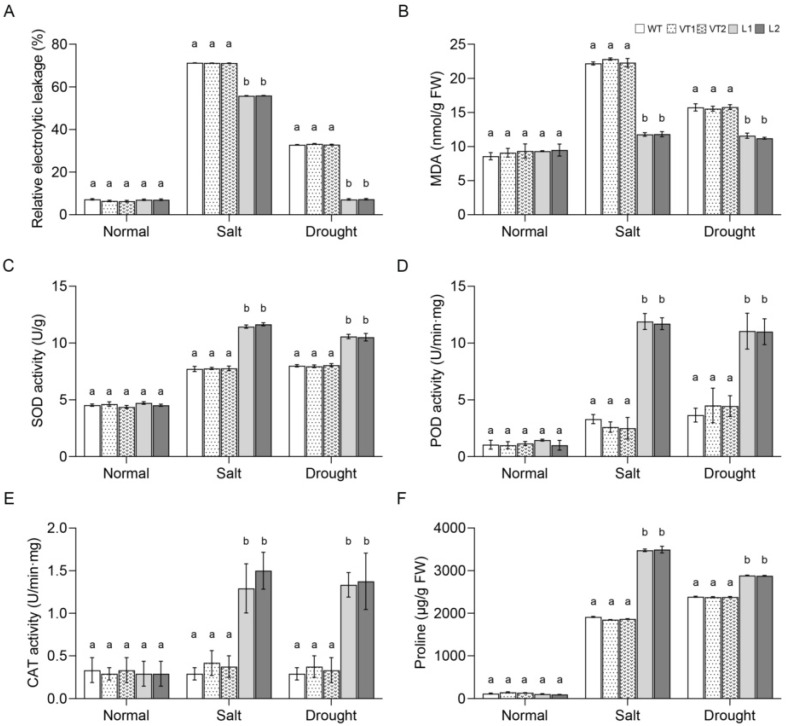
Physiological analyses of *Arabidopsis* under drought and salt stress. (**A**) The relative electrical conductivity; (**B**) MDA content; (**C**) SOD activity; (**D**) POD activity; (**E**) CAT activity; (**F**) Proline content of wild and transgenic *Arabidopsis* under normal conditions and stress. The data were means ± SD from three independent replications. Different lowercase letters indicate significant differences between different columns under the same treatment conditions (*p* < 0.05).

Plants suffer varying degrees of damage to their membrane systems in adverse environments. Relative conductivity correlates with the amount of electrolyte leakage, allowing an indirect measure of plasma membrane permeability, and the amount of MDA in plants can reveal the extent of membrane lipid peroxidation and thus indirectly detect plant stress tolerance. The relative conductivity and MDA content of all lines of *Arabidopsis* were low in normal culture; when subjected to salt and drought stress, the relative conductivity and MDA content of all lines increased to varying degrees and were lower in the L1 and L2 lines than in the WT, VT1 and VT2 lines (Figure 7A,B). These results indicated that the *SlNAC10* transgenic *Arabidopsis* membrane system was less damaged under stress treatment.

Plants produce large amounts of reactive oxygen species under adverse conditions, which can be scavenged by antioxidant enzymes. Based on an analysis of antioxidant enzyme activities, it was found that SOD, POD, and CAT activities were low in Arabidopsis of all plants grown in normal culture. When salt and drought stresses were encountered, SOD, POD, and CAT activities increased to different degrees in all *Arabidopsis* lines. Additionally, SOD, POD, and CAT activities were significantly higher in the L1 and L2 lines than in the WT, VT1, and VT2 lines (Figure 7C–E).

Proline is one of the most important osmoregulatory substances in plants. In our study, there was no statistically significant difference between the lines in the proline levels in *Arabidopsis* plants grown in normal culture. However, the proline content in all *Arabidopsis* lines increased to different degrees after salt and drought stress treatments, with the L1 and L2 lines having significantly higher proline content compared to the WT, VT1, and VT2 lines (Figure 7F), meaning that *SlNAC10* was able to improve the tolerance of *Arabidopsis* to adversity by accumulating proline. It is worth noting that proline accumulation is not the only mechanism that enhances plant stress resistance; there may be other mechanisms that act synergistically with proline and this will be the direction of our future research.

### 2.5. SlNAC10 Upregulates the Expression of Proline Synthesis-Related Enzyme Genes

**Figure 8:**
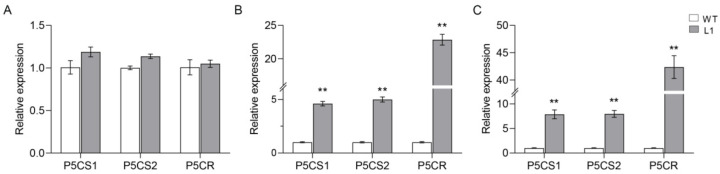
Expression of proline synthesis-related enzyme genes in *Arabidopsis thaliana* leaves under different treatment conditions. Relative expression levels of each gene (**A**) under unstressed conditions; (**B**) under NaCl stress; (**C**) under PEG stress. The data were means ± SD from three independent replications. The asterisks above the columns indicate a significant difference compared to WT (** *p* < 0.01).

As a result of our study, we found that proline content was increased in *SlNAC10* transgenic *Arabidopsis thaliana* under adverse conditions and the promoters of the proline synthesis-related enzyme genes *P5CS1*, *P5CS2*, and *P5CR* all contained NAC binding sites (CGTG/A). Using qRT-PCR, we examined the expression of the *AtP5CS1*, *AtP5CS2*, and *AtP5CR* genes in the wild-type (WT) and transgenic plants (L1). The expression of the *AtP5CS1*, *AtP5CS2*, and *AtP5CR* genes remained essentially the same in the WT and L1 *Arabidopsis* plants under unstressed treatments, whereas the expression of each gene was higher in the L1 lines than in the WT lines under salt and drought stress, with the highest expression of *AtP5CR* (Figure 8). These results indicate that the expression of the *AtP5CS1*, *AtP5CS2*, and *AtP5CR* genes is induced by salt and drought in *SlNAC10* transgenic *Arabidopsis*.

**Figure 9:**
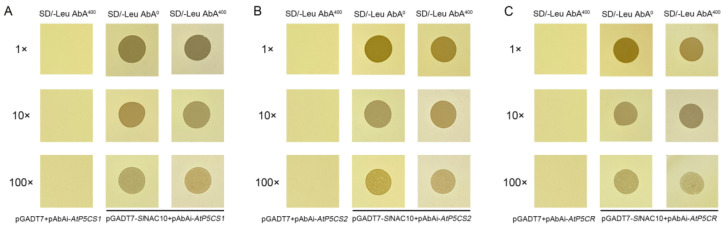
*Sl*NAC10 transcription factor binds to synthesis-related enzyme gene promoters. *Sl*NAC10 transcription factor binds to (**A**) AtP5CS1 promoter, (**B**) AtP5CS2 promoter, (**C**) AtP5CR promoter.

This was followed by a rapid examination of protein–DNA interactions using a yeast single-hybrid assay. Yeast growth can be inhibited by Aureobasidin A (AbA) and the pAbAi vector contains the AUR1-C and URA3 reporter genes. If *SlNAC10* can bind to the bait element inserted at the pAbAi multiple cloning site, the AUR1-C reporter gene can be activated to allow the yeast to grow on an AbA-containing medium. In order to prevent the leakage of other yeast and reporter genes from affecting the results of the experiment, the AbA concentration that inhibited the growth of the bait yeast was screened and the final AbA concentration of 400 ng/mL was determined as the background expression level for the bait yeast. The constructed pGADT7-*SlNAC10* recombinant vector and pGADT7 were then transferred into each bait yeast receptor cell and coated on different AbA concentrations of the medium. The results showed that the pGADT7-*SlNAC10* vector could grow on the SD/-Leu medium at AbA concentrations of 0ng/mL and 400ng/mL, whereas the pGADT7 empty bait yeast strain failed to grow on the SD/-Leu medium at AbA concentrations of 400 ng/mL, demonstrating that *Sl*NAC10 was able to bind to the AtP5CS1, AtP5CS2, and AtP5CR promoters (Figure 9).

## 3. Discussion

NAC transcription factors are one of the largest families of transcription factors in plants and play an important role in the transcription regulation of plant adaptation to environmental stresses. In our previous study, we found more than 20 NAC genes (*SlNAC*s) in *S. liaotungensis*, of which *SlNAC1*, *SlNAC2*, *SlNAC7,* and *SlNAC8* have all been shown to play important roles in the adaptation to abiotic stresses [33,34,35,36]. In this study, we cloned a new NAC transcription factor, *SlNAC10*, from *S. liaotungensis* and demonstrated that this gene improved tolerance to salt and drought stress in transgenic *Arabidopsis* by directly regulating proline synthesis-related enzyme gene expression and proline synthesis.

The full length of the *SlNAC10* cDNA was obtained from the EST sequence in the transcriptome database. The multiple sequence alignment showed that the N terminus of the gene had high similarity to the N terminus of other NACs and had a conserved NAC structural domain, which was identified as a NAC transcription factor. By constructing a phylogenetic tree, *Sl*NAC10 was shown to be highly similar to *At*NAC19, *At*NAM, and *At*ATAF (Figure 1), with *At*NAM having a role in stress resistance [37], and it is hypothesized that *SlNAC10* may also have a role in the response to stress resistance.

Typical transcription factors have a nuclear localization signal. For example, the MaNAC1-MaNAC5 proteins in bananas were distributed in the nucleus and MaNAC6 was distributed throughout the entire cell [38]. The *ChNAC1* transcription factor of the Chinese dwarf cherry (*Cerasus humilis*) was localized to the cell nucleus [39]. The subcellular localization analysis in this study showed that *Sl*NAC10-GFP was distributed in the nucleus (Figure 3). Because transcription factors often have transcription activation functional regions that regulate downstream genes at the transcription level, many studies have examined the transcription activation activity of the NAC transcription factors, for example, the *Brassica napus* protein BnNAC2 has transcription activation activity but BnNAC5 has no transcription activation activity [40]. A novel *Miscanthus* NAC gene *MlNAC12* has a transactivation activity in the C-terminus [41]. Our study of the transactivation activity of *Sl*NAC10 revealed that the C-terminus of *Sl*NAC10 has transcriptional activation activity, demonstrating that *Sl*NAC10 is a transcription factor and plays a role in the transcription of genes (Figure 4).

An increasing number of studies have reported the involvement of NAC transcription factors in plant responses to abiotic stresses, including salt, drought, and cold. For example, *GmNAC06* transcript levels were the highest in soybean cotyledons and *GmNAC06* expression was significantly increased in leaves and roots after induction by salt, ABA, cold, and PEG [42]. The expression level of *TaNAC29* was higher in wheat leaves and the transcription of *TaNAC29* increased after salt, PEG6000, H_2_O_2,_ and ABA treatments [43]. In this study, *SlNAC10* was induced by various abiotic stress treatments and the expression was significantly different in the roots, stems, and leaves of *S. liaotungensis* (Figure 2). In addition, we focused on the role of *SlNAC10* in response to salt and drought stresses. Longer primary root lengths and higher survival rates in *SlNAC10* transgenic *Arabidopsis* under salt and drought conditions were observed, presumably because *SlNAC10* enhanced the ability of transgenic *Arabidopsis* to withstand stress (Figure 5). This is similar to the way that *OsNAC6*-overexpressing rice lines show higher drought tolerance and *OsNAC6* improves adaptation to adversity by increasing the number of roots and expanding the root diameter [44]. *RhNAC31* overexpression is associated with increased cold tolerance in *Arabidopsis*, conferring a higher survival rate [45]. We subsequently found that the leaves of *SlNAC10* transgenic *Arabidopsis thaliana* under salt and drought stress treatments retained more green color to maintain photosynthetic activity, resulting in a higher survival rate for transgenic *Arabidopsis*, whereas non-transgenic *Arabidopsis* wilted, yellowed, and died. The Fv/Fm values of *Arabidopsis* were measured under stress treatment and it was found that the transgenic *Arabidopsis* maintained higher photosynthetic activity under stress conditions, indicating that the *SlNAC10* gene contributes to plant stress tolerance (Figure 6). The strength of photosynthesis directly affects the growth and development of *Arabidopsis* plants. The magnitude of the photosynthetic capacity is limited by its own photosynthetic system, for example, the transgenic lines of banana cultivar *Rasthali* overexpressing *MusaNAC042* can maintain higher chlorophyll levels and better Fv/Fm values in stressful environments [46]. The Fv/Fm images and chlorophyll content revealed that the WT plants were significantly damaged compared with the *CmNAC1*-EE plants under salt stress [47].

In an adverse environment, the relative conductivity and MDA content can reflect the integrity of the plasma membrane. The antioxidant enzyme system is the mainstay of enzymatic defense in an adverse environment and is responsible for the removal of reactive oxygen species from the body. In addition, stress treatments can cause an increase in osmoregulatory substances and proline is one of the most important osmoregulatory substances in the plant. Studies have shown that transgenic *Arabidopsis* with high expression of *TtNAC2A* has greater water retention, weaker oxidative stress, lower electrolyte leakage, and higher proline content compared to wild-type plants [48]. Chrysanthemum (*Dendranthema morifolium*) *DgNAC1* overexpressing showed lower electrical conductivity under salt stress, whereas the transgenic chrysanthemum showed reduced accumulation of MDA and reactive oxygen species; increased SOD, CAT, and POD activities; and increased proline content, enhancing the resistance of chrysanthemum to salt stress [49]. The *LpNAC13* overexpression plants had increased antioxidant enzyme activities and proline content, and decreased MDA content under salt conditions [50]. In our study, salt and drought stresses in all lines of *Arabidopsis* caused an increase in MDA content and relative electrolyte leakage in the plants but to a lesser extent in the transgenic *Arabidopsis*, indicating that the membranes of the transgenic *Arabidopsis* with the *SlNAC10* gene were less damaged and more resistant to the adverse environment. Furthermore, the transgenic *Arabidopsis* had higher antioxidant enzyme activity and proline content under stress conditions, which fully coordinated the enzymatic defense system in the plant and regulated the content of osmoregulatory substances, and could better adapt to the adverse environment, which was also a reflection of the enhanced stress resistance of the transgenic *Arabidopsis* with the *SlNAC10* gene (Figure 7).

Plants accumulate and produce large amounts of proline under adverse environmental conditions and the accumulation of proline reflects the plant’s resistance to unfavorable conditions. Other studies have found that under salt stress, proline content was increased in *ScPIP2-1*-overexpressing *Arabidopsis* and the *AtP5CS1* and *AtP5CS2* genes were highly expressed, demonstrating the enhanced salt tolerance of transgenic *Arabidopsis* [51]. Overexpression of the cotton *GhMYB4* gene leads to proline accumulation through the upregulation of *AtP5CS* and *AtP5CR* expression, ultimately leading to enhanced salt tolerance and drought resistance in transgenic *Arabidopsis* [52]. The yeast single-hybrid assay can measure the interaction between the DNA and plant transcription factors. For example, the ability of CmCyC2c to bind to the ClCyC2f promoter was demonstrated by a yeast single hybridization assay, providing clues that CmCYC2-like transcription factors may interact with each other or bind to the promoter to regulate floral symmetry development in *C. morifolium* [53]. Yeast single hybridization also confirmed the binding of CsATAF1 to the CsABI5, CsCu-ZSOD, and CsDREB2C promoters, providing evidence that CsATAF1 may be a transcriptional activator regulating CsCu-ZnSOD, CsABI5, and CsDREB2C [54]. In this study, we found that *SlNAC10* could enhance the resistance to abiotic stresses of transgenic *Arabidopsis* by up-regulating the expression of proline synthesis-related enzyme genes (Figure 8). In addition, *Sl*NAC10 was suggested to bind to the promoters of the *AtP5CS1*, *AtP5CS2,* and *AtP5CR* genes and regulate the transcription of their downstream genes by a yeast single hybridization assay (Figure 9).

In summary, our study shows that *SlNAC10* can improve the tolerance of transgenic *Arabidopsis* to salt and drought stresses. Therefore, we believe that *SlNAC10* has a potential utility as a natural novel resource for the cultivation of new crop varieties in saline or arid regions.

## 4. Materials and Methods

### 4.1. Plant Material and Treatment

*Suaeda liaotungensis* leaves and seeds were collected in Yingchengzi, Dalian, Liaoning, China. The leaves were frozen in liquid nitrogen and stored in a refrigerator at −80 °C for the cloning of the *SlNAC10* gene. Seeds were stored in a refrigerator at 4 °C and seedlings were raised in a greenhouse (25 °C, 16 h of light/8 h of dark, 50–65% humidity) for the study of the *SlNAC10* expression profile.

*Arabidopsis thaliana* (Columbia ecotype) was used for the gene transformation and phenotypic analysis. *Arabidopsis* seeds were grown on MS solid medium for 10 days at 22 °C with constant light, and seedlings were transplanted in a 1:1 soil mixture of humus and vermiculite and cultured in a greenhouse (23 °C, 16 h of light/8 h of dark).

### 4.2. Cloning and Sequence Analysis of the SlNAC10 Gene

RNAiso Plus (Takara, Beijing, China) was used to extract RNA from *S. liaotungensis* leaves, and total RNA was used as a template for reverse transcription into cDNA using PrimeScript^®^ RT Master Mix Perfect Real Time (Takara, Beijing, China). To obtain a partial fragment of *SlNAC10*, primers (Table S1, EST-F and EST-R) were designed based on the EST sequence of *SlNAC10*. The primers (Table S1, 3′-Outer, 3′-Inner and 5′-Outer, 5′-Inner) were then used to perform 3′, 5′ RACE to obtain the cDNA sequence of *SlNAC10*. Finally, the full length of *SlNAC10* was obtained by amplification using primers (Table S1, full length-F and full length-R) and was cloned into a pEASY-T1 vector (TransGen Biotech, Beijing, China).

We performed a BLAST search of the NCBI database (http://www.ncbi.nlm.nih.gov) to identify the sequences homologous to *SlNAC10* and performed multiple sequence comparisons using DNAMAN software (version 6.0.40, LynnonBiosoft, CA, USA). Using MEGA software (version 7.0, Mega Limited, Auckland, New Zealand), a phylogenetic tree was then constructed from the NACs of other plants, which were found to have high similarity to *Sl*NAC10. DNAMAN was used for the multiple sequence alignment and the NJ method in the MEGA 7.0 software was used to construct the evolutionary trees.

### 4.3. Analysis of SlNAC10 Gene Expression Profile

The *S. liaotungensis* seeds were sown in moist soil and kept in a greenhouse (25 °C, 16 h of light/8 h of dark, 50–65% humidity) and then re-cultured in a 1/10 MS liquid medium when 6–8 true leaves had grown. When the growth was good, the roots, stems, and leaves of unstressed *S. liaotungensis* were collected and then the other seedlings were treated with 200 mmol/L NaCl, 4 °C, 20% PEG (polyethylene glycol), and 100 μmol/L ABA, and the leaves were collected at 0, 0.5, 1, 3, 6, and 12 h after treatment.

*S. liaotungensis* RNA was extracted using RNAiso Plus (Takara, Beijing, China) and cDNA was obtained using TransScript^®^ One-Step gDNA Removal and cDNA Synthesis SuperMix. The primers (Table S1, 10-qF and 10-qR) and SYBR^®^ Premix Ex Taq™ (Perfect Real Time) kit were used to perform real-time fluorescence quantitative PCR in a Thermal Cycler Dice Real Time System TP800 (TaKaRa, Beijing, China). *SlActin* (GenBank No. JX860282.1) served as an internal reference and was amplified using primers (Table S1, SlActin-qF and SlActin-qR). Relative gene expression was calculated using the 2^−ΔΔCT^ method [55].

### 4.4. Subcellular Localization Analysis of SlNAC10

Using pEASY-T1-*SlNAC10* as a template, the open reading frame (ORF) fragment of *SlNAC10* was PCR amplified and cloned into a pEGAD-GFP vector (BioVector, Beijing, China) to construct the pEGAD-*SlNAC10*-GFP fusion expression vector. Transformation of the onion epidermal cells was completed using a gene gun (PDS-1000, China) and the green fluorescent protein fluorescence was observed on a laser confocal microscope (Zeiss LSM700; Zeiss, Jena, Germany) after the cultured onion epidermis.

### 4.5. Transcriptional Activation Analysis of SlNAC10

Using pEASY-T1-*SlNAC10* as a template, the full length and two truncated sequences of the *SlNAC10* gene were amplified and inserted into a PGBKT7 vector (Clontech, San Jose, CA, USA) to generate the pGBKT7-*SlNAC10*-F (full length), pGBKT7-*SlNAC10*-N (N-terminal), and pGBKT7-*SlNAC10*-C (C-terminal) yeast expression vectors. To screen for positive clones and determine *β*-galactosidase activity, the above yeast expression vector was transformed into yeast AH109 (Clontech, San Jose, CA, USA) and cultured in SD/-Trp and SD/-Trp-His-Ade media (Yeast Protocol Handbook; Clontech, San Jose, CA, USA). Relative gene expression was calculated using the 2^−ΔΔCT^ method [55].

### 4.6. Growth Assay of SlNAC10 Transgenic Arabidopsis under Salt and Drought Stress

The coding region of *SlNAC10* was cloned into a pBI121 vector. The pBI121-*SlNAC10* and pBI121 were transformed into *Agrobacterium tumefaciens* GV3101 by the freeze-thaw method and then into *Arabidopsis thaliana* (Columbia ecotype) by the floral dipping method, respectively. Homozygous transgenic *Arabidopsis* lines were obtained by screening on an MS medium containing 100 mg/L kanamycin and determined by PCR and semi-quantitative RT-PCR. Relative gene expression was calculated using the 2^−ΔΔCT^ method [55].

Subsequently, the measurements of the primary root length and survival ratio of *Arabidopsis thaliana* under salt stress and drought stress were carried out using T3 homozygous transgenic *Arabidopsis* and wild-type (WT) plants. Seeds of the above *Arabidopsis* lines were sown in MS solid medium with 0, 150 mM NaCl, and 200 mM D-mannitol. After 7 d, plants of equal growth were transferred to square Petri dishes containing MS + 150 mM NaCl and MS + 200 mM D-mannitol medium and cultured vertically for 8 d. The main root lengths were measured. *Arabidopsis thaliana* cultured normally for one week with almost the same growth were treated with stress (salt stress was applied by pouring 1 L of 300 mM NaCl solution every 7 d for 21 d and after 21 d of drought stress, the plants were re-watered for 3 d). The number of survivors of each plant was then counted and the survival rate was calculated.

### 4.7. Determination of Physiological Indicators of SlNAC10 Transgenic Arabidopsis Thaliana

One-week-old wild-type and transgenic *Arabidopsis* seedlings were selected and treated with salt stress (1 L of 300 mM NaCl solution every 7 d for 21 d) and drought stress (no watering for 21 d and re-watering for 3 d). The Fv/Fm and photochemical parameters were measured every 7 d starting from before the stress treatment using a chlorophyll fluorescence imager (IMAGING-PAM, Walz GmbH, Bad Waldsee, Germany) to take photographs.

The above well-grown *Arabidopsis* lines were subjected to 15 d of salt stress (1 L of 300 mM NaCl solution every 7 d) and drought stress (no watering). The relative conductivity in leaves was determined using a conductivity meter (DDS-11A, Shanghai Pengshun Technology Co., Ltd., Shanghai, China) [56]. In addition, the MDA content and the CAT, SOD, and POD activities, as well as the proline content, of the leaves were also measured [26].

### 4.8. Expression of P5CS1, P5CS2, and P5CR in Arabidopsis thaliana Overexpressing the SlNAC10 Gene

When the *Arabidopsis* seedlings of the WT and trans-*SlNAC10* type had grown 10 leaves, they were transferred, respectively, to a one-half MS liquid medium, one-half MS liquid medium containing 200 mM NaCl, and one-half MS liquid medium containing 10% PEG for 6 h. The true leaves of the seedlings from different plants were then cut and RNA was extracted using RNAiso Plus (TAKARA, Beijing, China). TransScript^®^ One-Step gDNA Removal and cDNA Synthesis SuperMix were used for reverse transcription. Primers (Table S1, AtP5CS1-qF, AtP5CS1-qR, AtP5CS2-qF, AtP5CS2-qR, AtP5CR-qF, AtP5CR-qR) for qRT-PCR were designed based on the sequences of *AtP5CS1* (GenBank: AT2G39800), *AtP5CS2* (GenBank: AT3G55610), and *AtP5CR* (GenBank: AT5G14800). *Atactin2* (GenBank: NM_112764) was used as the internal reference gene and primers (Table S1, AtActin-qF and AtActin-qR) were designed based on its sequence. Relative gene expression was calculated using the 2^−ΔΔCT^ method.

### 4.9. Analysis of the Interaction between SlNAC10 and the P5CS1, P5CS2, and P5CR Promoters

The core regions of the *AtP5CS1*, *AtP5CS2*, and *AtP5CR* promoters were predicted using BDGP: Neural Network Promoter Prediction online software, and then the promoter core regions were amplified using primers (Table S1, AtP5CS1-F, AtP5CS1-R, AtP5CS2-F, AtP5CS2-R, AtP5CR-F, AtP5CR-R). Yeast bait vectors pAbAi-*P5CS1*, pAbAi-*P5CS2*, and pAbAi-*P5CR* were constructed and linearized, transformed into Y1H Gold receptor cells using the Quick Easy Yeast Transformation Mix kit, and then assayed for background expression levels of the bait yeast reporter genes. Relative gene expression was calculated using the 2^−ΔΔCT^ method [55].

The pGADT7-*SlNAC10* vector was constructed by amplifying the *SlNAC10* gene using primers (Table S1, SlNAC10-F and SlNAC10-R) and cloning it into the pGADT7 vector. The bait yeast strain was prepared as the receptor cells, and the pGADT7-*SlNAC10* plasmid was transformed into the receptor cells of the bait yeast strain and coated in an SD/-Leu medium with an AbA concentration of 0 ng/mL and AbA concentration of 400 ng/mL, which was incubated at 30 °C for 3 to 5 days at a constant temperature with pGADT7 as the control, to verify whether the transcription factor *SlNAC10* interacts with the promoters AtP5CS1, AtP5CS2, and AtP5CR.

### 4.10. Statistical Analysis

Statistical analysis was performed by ANOVA using SPSS 13.0 software (SPSS, Inc., Chicago, IL, USA) and *p* < 0.05 was considered significant. Each experiment was performed in triplicate (transcriptional analysis *n* = 3; primary root length and survival analysis *n* = 60; physiological analysis *n* = 10).

## 5. Conclusions

**Figure 10:**
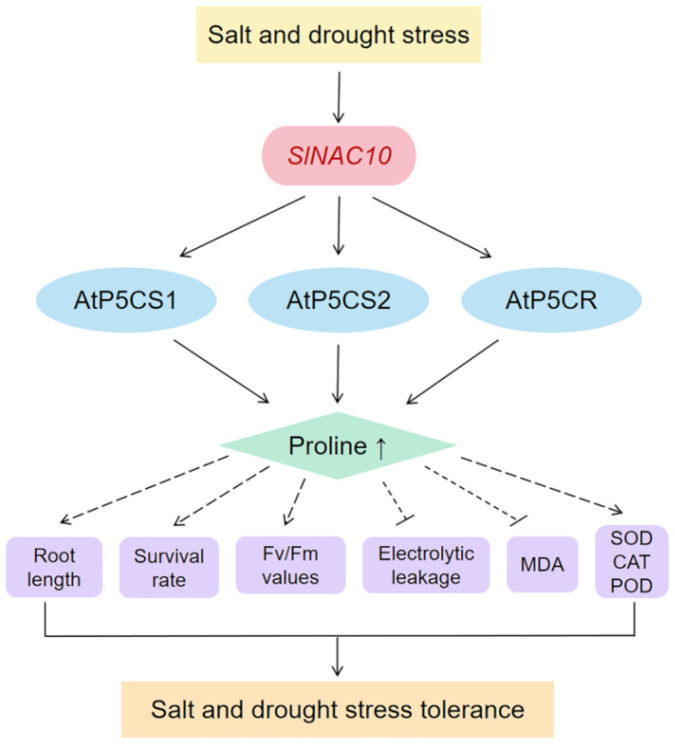
Technology roadmap of this study.

In this study, a new NAC transcription factor gene, *SlNAC10*, was cloned and identified as a transcription activator from the halophyte *S. liaotungensis*. Through a comprehensive analysis of *SlNAC10* in *S. liaotungensis* and transgenic *Arabidopsis thaliana* at the molecular, morphological, and physiological biochemistry levels, *SlNAC10* was confirmed to participate in abiotic stress responses and enhance salt and drought tolerance in plants. Proline is an important osmotic regulator in plants and can improve plant resistance under stressful conditions. Our study revealed that the proline content in *SlNAC10* transgenic *Arabidopsis* was increased and the expression of proline synthesis-related enzyme genes (*AtP5CS1*, *AtP5CS2*, and *AtP5CR*) were upregulated under salt and drought stress. Moreover, *Sl*NAC10 can bind to the AtP5CS1, AtP5CS2, and AtP5CR promoters and regulate the transcription of their downstream genes. Our results demonstrate that *SlNAC10* directly regulates the expression of proline synthesis-related enzyme genes and increases the proline synthesis, thus enhancing the salt and drought tolerance in plants. Therefore, we believe that *SlNAC10* can be studied in more depth to produce new varieties of stress-resistant crops (Figure 10).

## Data Availability

Data are contained within the article or Supplementary Materials.

## Supplementary Materials

The following supporting information can be downloaded at: https://www.mdpi.com/article/10.3390/ijms23179625/s1.
