# Supplementary material for: A Transcription Factor SlNAC10 Gene of Suaeda liaotungensis Regulates Proline Synthesis and Enhances Salt and Drought Tolerance
Source: Int J Mol Sci. 2022 Aug 25;23(17):9625. doi: 10.3390/ijms23179625 (PMC9455740; doi:10.3390/ijms23179625)
Supplement: Supplementary file 1 [file ijms-23-09625-s001.zip › ijms-1853761-supplementary.pdf]

Supplementary Materials

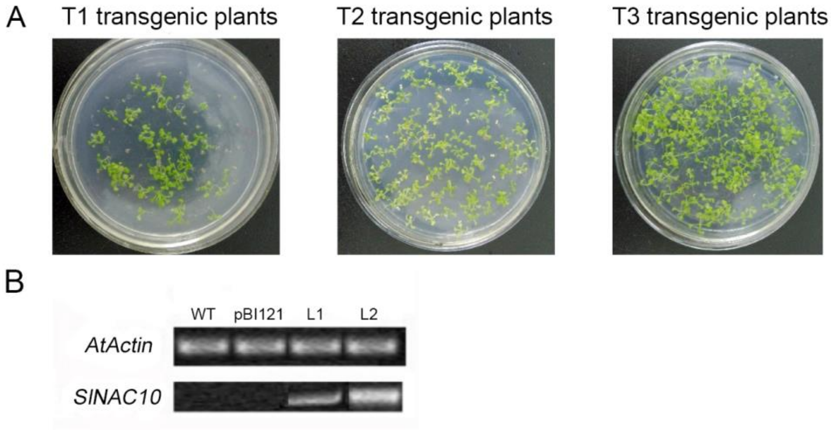

Figure S1. Kanamycin screening and RT-PCR of transgenic *Arabidopsis*. (A) Screening of transgenic *Arabidopsis*. (B) RT-PCR of wild-type *Arabidopsis*, pBI121 transgenic *Arabidopsis* and pBI121-*SlNAC10* transgenic *Arabidopsis* (L1, L2 lines). *AtActin* is *Actin* gene in *Arabidopsis*, used as the internal reference gene.

Table S1. The primers sequences used for SlNAC10 study.

EST-F	5′-TGGGAGTTCAAGAGAAAAAG-3′
EST-R	5′-GTGATGATGATGATGAGGAT-3′
5′-Outer	5′-ATCCACCTGTGCTGTGCTCCCTAC-3′
5′-Inner	5′-ACCTGTGCTGTGCTCCCTACTTGC-3′
3′-Outer	5′-GCCACGGGAACAGATAAAGT-3′
3′-Inner	5′-GCACGAATACCGTCTCACTG-3′
full length-F	5′-CTCTCAACAAAAACAAAAAC-3′
full length-R	5′-TCTCATCAGAGCATGAAA-3′
10-qF	5′-AGGCACTTGTGTTCTACGTTGGT-3′
10-qR	5′-GTGCTGTGCTCCCTACTTGCT-3′
SlActin-qF	5′-ATCCCAAGGCTAATCGTGAAAA-3′
SlActin-qR	5′-CACCATCACCAGAGTCCAACA-3′
ORF-F	5′-ggGAATTCATGGGAGTTCAAGAGAAAGA-3′ (EcoR Ⅰ)
ORF-R	5′-ggGGATCCTCTCATTCTACGGTCTGTA-3′ (BamH Ⅰ)
10-F	5′-gggaattcATGGGAGTTCAAGAGAAAGA-3′ (EcoR Ⅰ)
10-R	5′- ggggatccTCTCATTCTACGGTCTGTA -3′ (BamH Ⅰ)
10-N-R	5′-ggggatccAGTGAGACGGTATTCGTGC -3′ (BamH Ⅰ)
10-C-F	5′-gggaattcGAACACAATCGCAAAACCGGT-3′ (EcoR Ⅰ)
121-F	5′-ggggatccGATTATGGGAGTTCAAGAG-3′ (BamH Ⅰ)
121-R	5′-gggagctcTCAAAATTGTTTTAGTTTCTTC-3′ (Sac Ⅰ)
AtActin-qF	5′- CGAGGCTCCTCTTAACCCAA-3′
AtActinqR	5′-ACCATCACCAGAATCCAGCA-3′
AtP5CS1-qF	5′-CTGCAGAGCAATGGAGTCAC-3′
AtP5CS1-qR	5′-ACTCCCATGTCGGTGAATGT-3′
AtP5CS2-qF	5′- CCAACATGGAAGTGCACACA-3′
AtP5CS2-qR	5′- TGCTTATTCCCACCTCAGCA-3′
AtP5CR-qF	5′-TCTCAATCGCCGTGATGTCT-3′
AtP5CR -qR	5′-TTCCAGCTGCAACAGAAACC-3′
AtP5CS1-F	5′-AGCTTATACACAGCGTACATTTTATATACACAGCGTACATTTTATC-3′ (Hind III)
AtP5CS1-R	5′-TCGAGATAAAATGTACGCTGTGTATATAAAATGTACGCTGTGTATA -3′ (Xho Ⅰ)
AtP5CS2-F	5′-AGCTTGCTCTTGTTGTCGCGTGTCCTGCTCTTGTTGTCGCGTGTCCTC-3′ (Hind III)
AtP5CS2-R	5′-TCGAGAGGACACGCGACAACAAGAGCAGGACACGCGACAACAAGAGCA-3′ (Xho Ⅰ)
AtP5CR-F	5′-AGCTTACTTTCACGAGTCGGCGTCGTATAATATCGTCGTATAATAC-3′ (Hind III)
AtP5CR-R	5′-TCGAGTATTATACGACGATATTATACGACGCCGACTCGTGAAAGTA-3′ (Xho Ⅰ)
SlNAC10-F	5′-ccggaattcATGGGAGTTCAAGAGAAAGA-3′
SlNAC10-R	5′-cgcggatccCTACGGTCTGTATCCAAAC-3′
